# An Eight-Week Randomized Controlled Trial of Active Mobilization of the Hamstrings for Non-Specific Low Back Pain and Musculoskeletal Discomfort during Prolonged Sitting among Young People: Study Protocol

**DOI:** 10.3390/jcm13144161

**Published:** 2024-07-16

**Authors:** Marta Kinga Labecka, Magdalena Plandowska, Aleksandra Truszczyńska-Baszak, Reza Rajabi, Maciej Płaszewski, Dorota Różańska

**Affiliations:** 1 Faculty of Rehabilitation, Jozef Pilsudski University of Physical Education, 00-968 Warsaw, Poland; aleksandra.truszczynska@awf.edu.pl; 2 Faculty of Physical Education and Health in Biala Podlaska, Jozef Pilsudski University of Physical Education, 21-500 Biala Podlaska, Poland; magdalena.plandowska@awf.edu.pl (M.P.); maciej.plaszewski@awf.edu.pl (M.P.); dorota.rozanska@awf.edu.pl (D.R.); 3 Department of Health and Sport Medicine, Faculty of Physical Education and Sport Sciences, University of Tehran, Tehran 1417614411, Iran; reza.rajabi@awf.edu.pl

**Keywords:** exercise, treatment, spine, posture, students, pain, hamstring muscles

## Abstract

Participants will be recruited from the Faculty of Physical Education and randomly assigned to either the hamstring stretching group or the control group with education only. The primary outcome measures will be pain intensity, musculoskeletal discomfort, and functional disability. Secondary outcome measures will be satisfaction with the intervention and flexibility of the hamstring. A total of 44 participants fulfilling the inclusion criteria will complete the study. As an increase in LBP frequency is observed, it seems justified to determine effective interventions for LBP and musculoskeletal discomfort in young people. The findings of this study will provide information about the effect of an 8-week intervention involving active hamstring flexibility exercises with hip flexion mobilization on the reduction of LBP and musculoskeletal discomfort during prolonged sitting in young adults. We hope this study will add to the development of ergonomic recommendations for young people with LBP.

## 1. Introduction

Decreased flexibility of muscles, poor posture, prolonged sitting, fatigue resulting in soft tissue exertion, lumbosacral overload, and increased muscle fiber tone lead to an imbalance in lumbopelvic rhythm [1,2,3]. Four muscle groups support the pelvis and keep it in its natural position, including the erector spinae, hamstrings, gluteus maximus, abdominals, and hip flexors [4,5]. Weakening of central stabilization forces the musculoskeletal system to activate other stabilization mechanisms in areas distant from the spine, such as hyperactivity of the hamstrings [1,6].

Restricted lower extremity muscle flexibility, including in the hamstring, iliopsoas, and quadriceps, and limited range of motion of the hip joint, might be risk factors for low back pain, LBP [7]. LBP is defined as pain in the posterior part of the body from the lower margin of the 12th ribs to the lower gluteal folds, without pain referred to one or both lower limbs, and lasting for at least 1 day [8]. LBP can be specific or non-specific. Specific pain is pain caused by a certain disease or structural problem in the spine, or pain radiating from another part of the body due to underlying conditions such as spondylolisthesis, stenosis, malignancy, or inflammatory processes [9]. We are interested in non-specific pain, which is pain that cannot be explained with reference to a specific disease or structural problem. The prevalence of LBP has increased significantly over the last twenty years, especially among young people [8].

Muscle flexibility is essential in sports and physical fitness [10]. The length of the hamstring influences the motion of the pelvis during hip movements, consequently influencing the angle of lumbar lordosis [11,12,13]. In most daily and sporting activities, the hamstring muscles are active, and that is why it is necessary to maintain their normal length. Hamstring stretching should create an opportunity to practice movement and develop correct spinal patterns during hip flexion and extension. Maintaining neutral spinal curvature during activities is one of the basic principles of protecting the lumbar spine [14].

In individuals with LBP, decreased flexibility of muscles is associated with low back pain [15,16,17,18]. Stretching exercises are important in preventing and treating LBP [3,19]. One important option is the adoption of an exercise protocol that combines active hamstring stretching with hip flexion mobilization and the development of the habit of correct hip flexion, protecting the lumbar spine. Existing treatments for LBP often focus on general flexibility and strengthening exercises without targeting specific muscle groups such as the hamstrings, in conjunction with hip flexion techniques. This study addresses this gap by proposing a targeted intervention that may offer a more effective approach to managing LBP and discomfort in young adults, a demographic increasingly affected by sedentary lifestyles and prolonged sitting.

This is a protocol of a study that would evaluate the effectiveness of 8 weeks of active hamstring stretching exercises combined with hip flexion mobilization in reducing LBP and perceived musculoskeletal discomfort during prolonged sitting in young adults. 

## 2. Methods

### 2.1. Study Setting and Design

This will be a single-blind randomized controlled trial. The study protocol has been approved by the Senate Commission of Research Studies Ethics Józef Piłsudski University of Physical Education in Warsaw [SKE 01-05/2023], and the study has been prospectively registered at ClinicalTrials.gov [NCT05995145]. The protocol was prepared according to the Standard Protocol Items: Recommendations for Interventional Trials (SPIRIT) guidelines [20] and complies with the latest version of the Helsinki Declaration [21]. Written informed consent for participation will be obtained from all the participants involved in the study. The study will be conducted at the Faculty of Physical Education and Health, Jozef Pilsudski University of Physical Education in Warsaw, Biala Podlaska, Poland. The Consolidated Standards of Reporting Trials (CONSORT 2010) statement will be followed for the reporting of this study [22]. 

### 2.2. Study Population

The participants will be recruited according to the following inclusion criteria: (1) students of physical education; (2) age 18–25 years (18–25 years old); (3) with non-specific LBP; (4) with pain that has lasted at least 3 months; (5) without a history of spinal surgery; (6) no hamstring stretching exercises in the last 6 months; and (7) with hamstring muscle tightness.

The exclusion criteria are as follows: (1) specific LBP; (2) other criteria preventing participants from adequate participation in the study, such as pregnancyor menstrual pain; (3) leg length discrepancy over 1 cm; (4) previous spinal surgery; (5) presence of any contraindication to exercise; or (6) medically verified chronic back disorders.

### 2.3. Sample Size

The necessary minimum total number of subjects (n = 44) was obtained using the G*power program assuming a medium-sized effect (d = 0.50) at a significance level of 0.05 and a statistical power of 0.85. The sample will be increased by 10% to compensate for possible dropouts (overall sample = 48 participants). 

### 2.4. Randomization

Eligible participants will be randomly assigned to one of two groups: the experimental group (education, hamstring stretching, core stabilization, hip flexion mobilization) or the control group (education without any specific exercises). The randomization will be carried out by a researcher external to the study using a Microsoft Excel software random number generator before the beginning of the main study. Concealed allocation will be achieved using opaque envelopes. After the baseline assessment, the researcher responsible for the intervention will open the envelope to identify the group to which the participant will be allocated.

### 2.5. Blinding

This will be a single-blinded trial. Baseline and post-intervention measurements and measurement assessments will be performed by an evaluator who is blind to the group. The statistician performing the statistical analysis will also be blinded to the allocation and treatment of groups. Due to the nature of exercise interventions, it will not be possible to blind the participants.

### 2.6. Intervention Protocol

#### 2.6.1. Experimental Group

The hamstring stretching exercise program will be conducted five times a week, 20 min per session, for 8 weeks. The exercise program will be supervised by an experienced physiotherapist (5 years of clinical experience).

The intervention will be divided into two parts. The first part will include rolling with a foam roller [23]. For the roller massage, the participants will have to sit on a mat with a roller placed under the leg (Table 1). The participants will have to move up and down to massage their thighs between the buttocks and the knee. The roller massage will be performed for 5 min. The second part will include active hamstring stretching combined with hip flexion mobilization and the maintenance of correct hip flexion, protecting the lower spine. The exercise protocol will include three exercises (Table 1). The session will consist of 3 sets of each exercise. All positions will be maintained for 30 s with 30 s of relaxation [24]. The level of difficulty (level of hip flexion) will be self-regulated by the participant based on their physical condition and discomfort [25]. Equipment used in the active hamstring flexibility exercises program will consist of a massage foam roller and a gym mat.

In the experimental intervention, the exercise program will be based on participant education, home-based individual exercises, and regular group meetings for check-ups by a physiotherapist according to a preset schedule.

The first week of the intervention will consist of 4 full days of participant education and home exercise instruction. Sessions will be supplemented with information about the anatomy of the lower spine and advice, including postural instructions and practical demonstrations of lifting, pushing, pulling, and other daily actions. Other weeks of the intervention will be dedicated to individually prescribed home exercises. 

The first check-up with the physiotherapist will take place 2 weeks after the start of the exercise program, to check that the participant is performing the exercises with the correct execution and regularity. The next check-up with the physiotherapist will take place 4 weeks after the start of the exercise program when the physiotherapist will re-check the exercises. The participants will record their exercise frequency on a pre-prepared diary-record sheet. 

#### 2.6.2. Control Group

Students assigned to the control group will receive an educational booklet containing information on the causes of low back pain, the anatomy of the spine and its relation to the muscular chain, and tips for spinal care during daily life activities. The control group will be encouraged to perform their regular baseline activities without the addition of a specific lower extremity flexibility program. Participants will also be contacted weekly by email and/or telephone. Participants will be asked to continue their daily routine and receive neither physical therapy treatment programs nor other medical care.

The control group participants will be advised to take part in the same hamstring stretching training as the intervention group in the evaluation session after the study is completed.

### 2.7. Assessment

Participants who fulfill the eligibility criteria, agree to participate, and sign a written informed consent form will be included in the study subject to the assessment. A blinded assessor-physiotherapist with five years of clinical experience will check the qualifying criteria. A baseline assessment will be conducted before participants are randomly assigned to groups. An outcome assessment will be collected at the start of the study and immediately after the 8-week intervention.

### 2.8. Pre-Intervention Data Collection Sheet 

Before random group assignment, participants will receive the survey containing questions about age, gender, current physical activity (number of training days and hours per week, sports discipline), history of illnesses, injuries, surgeries due to spinal ailments, LBP physiotherapy and confirmed by which medical doctor, and body posture defects. A questionnaire will also be used to collect data about the experience of LBP within the last three months. Individuals who respond positively (“yes”) to the question “Have you experienced low back pain for the last three months?” will answer the question on the frequency of LBP (rarely, a few times per week, often, or constantly) and the types of situations in which LBP occurred, increased, or worsened.

### 2.9. Outcome Measures

Primary outcome measures (average pain intensity, functional disability, perceived musculoskeletal discomfort during prolonged sitting) will be collected at baseline and immediately after the 8-week intervention, and the global perceived improvement will be assessed immediately after the 8-week intervention. The secondary outcome measure (flexibility of the hamstring) will be collected after the 8-week intervention. Table 2 shows the distribution of the different measures across the timepoints during the study.

### 2.10. Primary Outcome Measures

1. Pain intensity

The Visual Analog Scale (VAS) will be used to assess average pain intensity graded from 0 (no pain) to 10 (unbearable pain). Mild pain or no pain will be defined as VAS 0–3, moderate pain as VAS 4–6, and severe pain as VAS 7–10 [26]. Participants will be asked to rate their maximum pain intensity from the last 3 months.

2. Functional disability

The Revised Oswestry Low Back Pain Disability Index (ODI) will be used to assess the level of functional disability resulting from LBP. The questionnaire consists of 10 items related to different aspects of function regarding activities of daily living. Each item will be scored from 0 to 5, with higher values representing greater disability. The total score will be multiplied by 2 and expressed as a percentage [27].

3. Perceived musculoskeletal discomfort during prolonged sitting

The Borg CR-10 scale will be used to assess perceived musculoskeletal discomfort during 1 h of sitting, classified as prolonged sitting. The Borg CR-10 scale is presented in such a way that test subjects can indicate in which parts of the body they feel discomfort (i.e., neck, shoulder, upper back, lower back, hip/thigh, and knee) and the degree of discomfort they feel (on a scale from 0 to 10, 0 being no discomfort and 10 being extremely uncomfortable) [28]. The scale and body chart will be determined according to a standardized Nordic questionnaire [29]. During university classes, the participants will be required to spend an hour sitting in a chair with a backrest. The initial sitting position will be characterized by hips and knees at 90 degrees of flexion and a neutral ankle position with feet in full contact with the floor. During the 1 h of sitting, the participants will be instructed to avoid talking and to maintain the assigned sitting posture as much as possible. They will be able to make small adjustments if they feel too much discomfort. The Borg CR-10 scale will be given to the participants to complete after the one-hour sitting session.

4. Global perceived improvement

The Global Perceived Effect (GPE) scale will be used to assess the global perceived improvement of the condition. The investigator will ask the participant to rate, on a numerical scale, how much their condition has improved or deteriorated since some predefined timepoint: 1 = completely recovered, 2 = much improved, 3 = slightly improved, 4 = not changed, 5 = slightly worsened, 6 = much worsened, and 7 = worse than ever. These ratings will be dichotomized into “improved” (GPE scores 1–2) and “not improved” (GPE scores 3–7) [30]. 

### 2.11. Secondary Outcome Measures

#### Flexibility of the Hamstrings

The flexibility of the hamstrings will be assessed using the single passive straight leg raise (SLR) test [31], measured in degrees by a goniometer. The SLR will be performed twice for each leg and the mean value of the two attempts will used for analysis [31]. A subject’s hamstring muscles will be considered tight (in a shortened position) if the SLR is ≤70° [32]. 

### 2.12. Timeline of the Study

We designed the timeline of the study using the SPIRIT framework (Table 1). At enrollment, the purpose of the study and research procedures will be explained to the participants. The pre-screening will address the specific inclusion and exclusion criteria for the study, basic demographic data, and the frequency of LBP. Participants who meet the inclusion criteria will be invited to attend a baseline assessment (Table 2). All the tests will be performed during morning hours in the laboratory. 

At the baseline assessment session, participants will be requested to complete the VAS, ODI, and Borg CR-10 scale. After completion of the baseline assessment, participants will be randomly assigned to one of two groups (experimental or control), and simultaneous implementation of the exercise program will commence. After 8 weeks, all assessment measures for all participants will be repeated in the same way as the baseline measurements (Table 2).

### 2.13. Data Analysis

The normality of the distributions will be checked using the Shapiro–Wilk test. Data will be presented as mean ± SD where appropriate. For variables that do not meet the criteria of normality of distribution, data will be presented as medians and quartile ranges. Lavene’s test will be conducted to verify the homogeneity of variances. The chi-square test will be used for categorical variables. The Mann–Whitney U test will be used to compare the groups. The mixed-design ANOVA with two factors (intervention, time) will be used. Tukey’s post hoc test will be used for detailed comparisons. Effect sizes will be assessed by partial eta squared (ANOVA) or the Glass rank-biserial correlation coefficient (the Mann–Whitney U test). The significance level will be set at *p* < 0.05.

## 3. Discussion

Stretching exercises play an important role in both the prevention and treatment of LBP [3,19]. Previously, a direct correlation between hamstring hyperactive strain and the severity of LBP was reported [7]. In contrast to other randomized control trials, our study has been designed to investigate the effect of active hamstring flexibility exercises and education regarding the use of the correct hip flexion technique (maintaining lumbar lordosis) to protect the lower spine on reduced LBP and perceived musculoskeletal discomfort during prolonged sitting. We believe that by improving the hip flexion technique, there will be a decrease in pain. Moreover, the suggested exercises may be more effective for pain and disability because they are focused on hip mobility and the stability of the lower back. If our hypothesis is proven, this may increase the treatment options for people with LBP. 

In 2019, the world faced the unforeseen COVID-19 pandemic, the effects of which adversely affected the health of the global population. Undoubtedly, the pandemic has contributed to the intensification of risk factors resulting in LBP. Interventions based on scientific evidence are extremely important because they will allow us to mitigate the effects of the unusual phenomenon of the pandemic by reducing the frequency and intensity of LBP. To the best of our knowledge, this problem has not yet been fully explored, especially among young people with LBP, and requires immediate attention to obtain the best available evidence and assess the effectiveness of planned interventions. 

Our research offers valuable evidence-based practices for managing and preventing low back pain. The specific research gap this study aims to fill is the lack of evidence on the effectiveness of active hamstring stretching exercises combined with hip flexion mobilization in reducing LBP and perceived musculoskeletal discomfort during prolonged sitting in young adults. Existing treatments for LBP often focus on general flexibility and strengthening exercises without targeting specific muscle groups such as the hamstrings, in conjunction with hip flexion techniques. This study addresses this gap by evaluating a targeted intervention that may offer a more effective approach to managing LBP and discomfort in young adults, a demographic increasingly affected by sedentary lifestyles and prolonged sitting.

We are aware that the protocol relies on self-reported measures for pain intensity, functional disability, and musculoskeletal discomfort, which may be subject to bias. Future studies should incorporate objective assessments such as electromyography (EMG) to measure muscle activity and flexibility more precisely. This tool can provide more accurate and reliable data on the physiological effects of the intervention. 

The study does not account for potential differences in response to the intervention between males and females. Gender-specific analyses cannot be performed due to the small sample size. In the future, we plan to perform gender-specific analyses to understand how males and females might respond differently to the intervention and tailor future protocols accordingly.

Future research should include a larger sample size to enable stratified analyses and explore potential gender differences. We are aware of this limitation in terms of the internal validity of our study [33]. In addition, with a total of 48 participants, the study’s findings may not be generalizable to the broader population of young adults with LBP. Larger-scale studies and long-term follow-up assessments are needed to confirm the results, enhance the generalizability of the findings, and explore the effects of the intervention across different populations, including various age groups and individuals with different levels of physical activity. 

In future studies, we plan to investigate different variations of the intervention, such as different durations, frequencies, and combinations with other therapeutic exercises, to identify the most effective protocols for reducing LBP and musculoskeletal discomfort. Despite these limitations, our research offers valuable evidence-based practices for managing and preventing low back pain. The specific research gap this study aims to fill is the lack of evidence on the effectiveness of active hamstring stretching exercises combined with hip flexion mobilization in reducing LBP and perceived musculoskeletal discomfort during prolonged sitting in young adults. Existing treatments for LBP often focus on general flexibility and strengthening exercises without targeting specific muscle groups such as the hamstrings, in conjunction with hip flexion techniques. This study addresses this gap by evaluating a targeted intervention that may offer a more effective approach to managing LBP and discomfort in young adults, a demographic increasingly affected by sedentary lifestyles and prolonged sitting.

## 4. Conclusions

The findings of this study will provide evidence of the effectiveness of an 8-week intervention involving active hamstring stretching combined with the maintenance of lordosis, core stability, and hip joint mobility to improve nonspecific LBP and musculoskeletal discomfort during prolonged sitting in young adults. We hope this study will help to develop ergonomic recommendations for young people suffering from this condition.

## Figures and Tables

**Table 1:** Exercises for active hamstring stretching with hip flexion mobilization.

1st part	
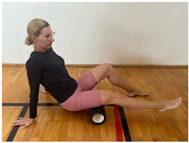	Foam roller massage. Starting position: sit on the mat with a straight spine, place the massage tool below the thigh, and roll the thigh between the buttock and the knee.
2nd part	
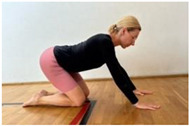 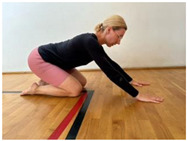	Learning the pattern of flexion in the hip joints. Starting position: kneeling supported. Activation of the core muscles, and awareness of setting the lumbar spine in a neutral position. Moving the hips towards the heels, keeping the spine in a neutral position. Notes: keep the lower spine stable and the pelvis in an anterior tilt.
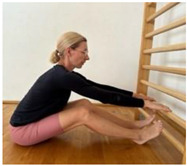 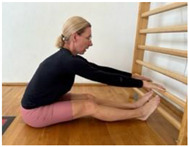	Active stretching of hamstrings with the development of the habit of correct hip flexion and knee extension. Starting position: sit with a straight back, knees slightly bent. Flex hips and straighten knees. Hold. Knee flexion. Increase hip flexion and knee extension. Notes: keep the lower spine stable and the pelvis in an anterior tilt. The degree of flexion in the hips is adapted to the participant’s abilities.
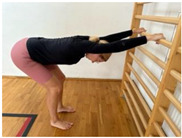 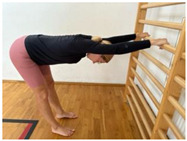	Active stretching of hamstrings with torso stabilization and correct hip flexion and extension; lumbar technique. Starting position: standing, facing the wall, legs slightly bent at the knees Hip flexion, then knee extension. Hold. Knee flexion. Hip flexion increase, knee extension. Notes: keep the lower spine stable and the pelvis in an anterior tilt. The degree of flexion in the hips is adapted to the participant’s abilities.

**Table 2:** SPIRIT table of enrollment, intervention, and assessment.

	Enrollment	Before Intervention	Randomization	Intervention
Timepoint	1st Month	2nd Month	2nd Month	3rd and 4th Months (8 Weeks)
Enrollment				
Eligibility pre-screening	+			
Informed consent	+			
Allocation			+	
Interventions				
Experimental and control group				+
Assessments				
Pain intensity		+		+
Functional disability		+		+
Perceived musculoskeletal discomfort		+		+
Global perceived improvement				+
Flexibility of the hamstring				+

## Data Availability

Not applicable. Data sharing does not apply to this article as no datasets were generated or analyzed during the current study.
